# Group-based exercise for Parkinson’s: a qualitative study of participants and partners’ perceptions of an exercise class delivered through a community-university collaboration

**DOI:** 10.1186/s12877-024-05061-7

**Published:** 2024-06-04

**Authors:** Anna Ferrusola-Pastrana, Christopher L Fullerton, Stephen N Meadows

**Affiliations:** 1 School of Sport and Exercise Sciences, University of Kent, Canterbury, UK; 2 University School of Health and Sport (EUSES), University of Girona, Salt, Spain; 3 Faculty of Health Sciences and Sport, University of Stirling, Stirling, FK9 7LA UK

**Keywords:** Community, Exercise, Focus groups, Parkinson’s disease, Qualitative research

## Abstract

**Background:**

Community-based exercise programmes (CBEPs) offer a practical and viable approach to providing people with Parkinson’s disease (PwP) the opportunity to exercise as an ancillary therapeutic benefit to pharmacological management. This study explores the perceptions of exercising participants (PwP) and non-participating partners involved in an exercise class delivered through a community-university partnership.

**Methods:**

Two separate focus group discussions were conducted: one with class participants (PwP: *n* = 7, H&Y scale I to III), and the other with non-participating partners of PwP (*n* = 4).

**Results:**

Thematic analysis of the data identified that a range of physical, psychological and social factors were perceived to influence engagement: (1) actively taking control, (2) exercise is medicine for the mind and body, and (3) a community working together to promote exercise for parkinson’s. Participants and partners felt that the support from the group, including the instructors and student volunteers, empowered and supported PwP to proactively self-manage their health, enjoy exercise in an inclusive group setting, and develop strong social connections with others in the local Parkinson’s community. Support to exercise from healthcare professionals was identified as both an enabler and barrier to participation.

**Conclusions:**

This study underscores the significance of a community-university partnership as a complementary therapeutic approach for PwP. It also provides critical reflections on its sustainability, including implications for how exercise is considered as medicine for PwP. Additionally, it offers practical recommendations to galvanise community participation and provide inclusive and viable exercise opportunities for PwP.

## Introduction

Non-pharmacological therapies, such as physical exercise training, can play an important part in overall disease management for people with Parkinson’s disease (PwP). Exercise is considered a low-cost, safe, and effective strategy that offers multiple biopsychosocial benefits [1–3]. Among various types of exercise suitable for PwP, multimodal (MM) exercise, which combines different components like aerobic, strength, flexibility, coordination, and balance exercises with cognitive training, has been extensively studied for addressing deficits presented in Parkinson’s disease (PD) and promoting exercise-induced neuroplasticity [4–9]. However, despite the strong evidence for MM exercise, its translation from trial to community settings requires a collaborative multidisciplinary approach to make it accessible, inclusive, enjoyable, and effective for PwP of all ages and diagnoses [10].

Multiple exercise approaches have been explored, yet determining the most effective modality and dosage (i.e., type, duration, frequency, and intensity) for PD symptoms management remains uncertain. Current guidelines recommend different exercise modalities including strength, balance, flexibility, and aerobic exercise at least thrice weekly, lasting ≥ 30 min at 40–60% heart rate reserve, or an intensity of 12–13 on a 20-point rating of perceived exertion (RPE) scale (with progression towards higher intensities) [11–13]. However, evidence suggests that long-term motor and cognitive benefits can still be obtained even with a lower frequency of training (i.e., once a week) [6].

Exercise interventions for PwP are typically delivered in healthcare (led by a clinical physiotherapist), community (group class), or home (individual programmes) settings. Whilst physiotherapists are well-placed to deliver such programmes [14], the current healthcare system in the United Kingdom (UK) lacks sufficient resources to provide intensive support on a large scale and over extended durations. Therefore, community-based exercise group sessions led by exercise professionals have been proposed as a solution to reduce healthcare costs [15, 16]. Moreover, a recent study described the positive experiences of PwP participating in a group balance exercise programme for 8 weeks, highlighting its safe and inclusive nature, which fulfilled their social and emotional needs [17, 18]. Exercising in groups provides PwP with opportunities for social engagement, breaks isolation, allows individuals to share coping strategies (helping to address daily struggles), and thereby promotes a more positive attitude towards life [17, 19, 20]. However, these findings primarily represent the experiences of PwP at an early stage of the disease within a specific community-based exercise programme (CBEP). It remains to be seen whether the reported group dynamics apply to longer-term (since most work has been conducted over the span of several weeks) and other types of exercise programmes.

Although delivering group-based exercise programmes (GBEPs) in the community can be challenging due to the lack of local expertise in PD and inadequate infrastructure, a collaborative partnership between local communities and academic institutions (i.e., community-university partnership approach) can be beneficial [21]. Similar to previous studies using a collaborative model [22], this approach allows for tailored, intergenerational support, facilitated by the presence of student volunteers, and collaborative decision-making processes to incorporate PwP’s needs [23]. Involving student volunteers offers several benefits, such as offering assistance and support within the class, providing co-learning and knowledge exchange, enriching the experience for both students and participants alike, and, altogether, fostering a sense of fellowship and community [24, 25].

The design and delivery of exercise training sessions, the social and physical environment, external support, and individual and group characteristics, are some of the most important factors influencing PwP’s engagement in exercise [26]. While previous work has explored the views of PwP, less is known about the perspectives of carers, who are often partners of PwP [27]. PwP tend to report positive views and perceived therapeutic benefits of exercise, including pleasure, well-being, increased confidence, and quality of life [28–30]. However, partners play an important part in the management of PD, and their views and experiences can contribute significantly to exploration of interpersonal processes involved in exercise participation [31]. By considering partners’ perspectives, we can provide a more holistic picture of the challenges and opportunities in the planning, design, and delivery of community-based exercise initiatives for PwP [29, 32].

Qualitative research involving participants and those supporting participation (e.g., spouses) can provide critical insights into the factors that support initiating and maintaining regular exercise. While previous studies have used such methods [26, 33, 34], there is limited understanding of the perceptions and experiences of PwP and their partners in exercise programmes delivered through a community-university partnership approach [26, 35]. Insights generated from these discussions can support the planning, feasibility, and long-term sustainability of exercise programmes for PwP. Therefore, the purpose of this study was to explore the perceptions and experiences of both PwP and their partners in relation to participating in (or supporting participation in) an exercise programme for PwP delivered through a community-university partnership. Additionally, the study aimed to use these findings as a tool for service improvement, provide feedback to the group, contribute to the broader Parkinson’s community, and offer practical recommendations to assist those delivering exercise programmes for individuals with PD.

## Methods

### Design and context

The design and analysis of the study were consistent with a pragmatist epistemology [36, 37]. In line with this epistemological view, the current study explored and analysed the perceptions and experiences of PwP who actively participated in a PD-specific exercise class [6], as well as the viewpoints of their non-participating partners. This pragmatic qualitative approach focused on learning from the participants and their partners and using this knowledge to create recommendations aimed at optimising future intervention delivery.

Data for this study were collected through two focus groups. This method offers a rich understanding of participants’ experiences and fosters spontaneous idea generation through participant interaction [38]. Additionally, focus group discussions can create a collective identity among participants and, thus, enable participants to connect their individual narratives with each other as well as the wider Parkinson’s community.

### Multimodal exercise class

The PD specific MM exercise programme was a longitudinal, community-based group exercise class. It commenced from late 2016 to early 2020 and consisted of a weekly circuit-based MM exercise programme in a community hall for 1 h per session. The exercises were structured in stations, emphasising meaningful, goal-related movements to address PD-specific characteristics like gait impairments and balance problems. Participants were encouraged to exert themselves at their perceived effort level, aiming for at least “somewhat hard” on the Borg 6–20 RPE scale. Sessions were delivered by two exercise professionals (Level 4 Specialist Exercise Instructor; Register of Exercise Professionals) and supported by undergraduate students from the University of Kent, who provided instructions and encouragement to maintain moderate to high intensity and proper technique (for more details see previously published work [6]).

### Participants

Following institutional ethical approval, two focus groups were held in March 2019 with 11 participants (PwP, *n* = 7; partners of PwP, *n* = 4) conveniently recruited from the exercise class. The recruited class participants (male, *n* = 7; age, 70 ± 9 years) had a diagnosis of mild to moderate idiopathic PD (Hoehn & Yahr [H&Y] scale I to III), disease duration of 4.6 ± 2.1 years and had been attending the exercise classes for an average of 2.0 ± 0.7 years, with an attendance rate of 74% ± 16% (ranging from 43 to 96%). Four partners of PwP also participated (female, *n* = 4; age 68 ± 6 years). The participants regularly accompanied their partners to the exercise class, waiting in a meeting room outside the hall. Participants were approached in person, two weeks prior to the focus group sessions. Participants were given a week to decide whether to take part after receiving an information sheet. Those who showed interest and were available to arrive early at the community centre were contacted by telephone to confirm their attendance. In addition, partners and attendees who arrived early on the day of the focus groups were invited to participate to avoid any selection bias.

### Focus group structure and questions

To encourage maximum participation with minimal time commitment, the focus groups were scheduled to coincide with the delivery of the exercise class. The second author, familiar to the group but not involved in the delivery of the exercise class, led both discussions. This approach created a comfortable environment, enabling participants to freely express their thoughts and experiences. Prior to the discussions, a brief introduction emphasised confidentiality, managed expectations, and allowed participants to reflect on their decision to participate.

Each focus group followed a similar structure. The first, comprising only class participants (PwP), met before the exercise class, while the second included non-participating partners ran concurrently with the class. This schedule allowed class participants to share information without their partners or someone without PD present, creating an environment where participants in both focus groups could feel comfortable sharing their feelings honestly [32]. Group durations varied, with the PwP discussion lasting 87 min and the non-participating partners discussion lasting 43 min. By scheduling the discussions before and during the class, we aimed to prevent disruption to participants’ exercise experience. Both discussions began with open-ended questions about the exercise class. Subsequent questions covered topics such as motivation for participation, perceived benefits and challenges of the class, factors influencing adherence, the structure and format of the exercise class, and feedback on the service (see Table 1). The facilitator used prompts to encourage further responses and clarify points, while participants were also encouraged to share any relevant information they deemed important [39].

**Table 1:** Focus group discussion topics and example questions

Focus Group Discussion Topics	Example of Questions
1. Exercise Class Experience	When you think about the exercise class, what is the first thing that pops into your mind?
2. Motivation for Participation	What was your [partner’s] motivation to join the class?
3. Benefits and Challenges	Have you noticed any positive effects on your [partner’s] health from the exercise?
4. Factors Associated with Engagement	Why do you think some of the local Parkinson’s support group do [not] attend the exercise classes?
5. Exercise Class Structure/Format	Do you think the exercises are appropriate for people with Parkinson’s?
6. Feedback on the Service	Are there any changes you would make to the exercise class to encourage greater participation?

### Data analysis

Our analysis of the data followed an iterative and reflexive process, using Braun and Clarke’s (2019) reflexive thematic analysis (RTA) method, which is accessible to researchers with limited qualitative research experience and aligns with our pragmatic approach [40]. We employed a team-based analysis approach [41], utilising the interdisciplinary skills and knowledge within our author team (i.e., clinical exercise physiology, biomedical sciences, and sport and exercise psychology).

Focus groups were Dictaphone recorded and transcribed verbatim with participants’ consent, then imported into NVivo 12 software for organising and analysing the qualitative data. Initial coding was conducted by a researcher with experience in qualitative research. Subsequently, to enhance the depth and richness of the analysis, the other co-authors actively participated in developing codes, defining themes, and shaping the final interpretation. This collaborative approach aimed to build insight and foster critical reflexivity by actively questioning each other’s knowledge construction [42]. This shift away from consensus between coders aimed to ensure that the resulting finalised thematic framework worked together to tell a rich story about the participants’ perceptions of the class. Notably, this approach recognised the evolving nature of qualitative analysis and the absence of a definitive endpoint, as discussed in the RTA framework [40, 43]. Regular formal team discussions were held to review the findings, providing opportunities for constructive challenges to the interpretation of the findings, sharing of different viewpoints, and identifying conceptual links between the themes. Findings are presented in relation to existing literature, emphasising a transparent and contextually grounded interpretation of the data.

## Results

Our analysis led to the creation and development of three distinct but interrelated themes: (1) actively taking control, (2) exercise is medicine for the mind and body, and (3) a community working together to promote exercise for Parkinson’s. These themes capture unique narratives that contribute to a nuanced understanding of the exercise class experience. The first theme describes the participants’ reasons for attending the class (from the perspective of both the participant and the partner), focusing on how individuals used the exercise class to support the management of Parkinson’s and how regularly attending provided social support and a network of people who share experiences. The second theme describes the perceived physical and psychological therapeutic benefits experienced by participants. The final theme describes the group coming together and the qualities of the environment that support the feeling of community and fellowship. Each theme is explored in more detail below.

### Theme 1: actively taking control

Exercise class attendees and partners discussed their reasons for joining and/or supporting the exercise class. Support from the university appeared to be a driving force behind attendees’ and partners’ involvement and engagement. For some class attendees, the exercise class played a pivotal role for participants in accepting, adapting to, and managing their Parkinson’s, whilst for others, joining an exercise class was seen as something that could have wider benefits to future PwP as well as research. The group, facilitated by both members and professionals, appeared to encourage and support individual and collective coping by nurturing togetherness and showing people that they are not alone. Partners expressed a desire to support their loved ones but also acknowledged the fatigue associated with these efforts, finding solace in the group’s social support.

***We’re actively engaged in fighting back***. The idea of “fighting back” against Parkinson’s is a well-known phenomenon in the Parkinson’s literature and practice [44–46], and is something that has also been reported in other chronic health conditions, such as stroke, cancer, and Alzheimer’s [47–49]. However, whereas “fighting back” is often associated with binary success outcomes (i.e., victory or defeat), for the attendees of this exercise class, it signifies something different. Rather than “winning” the fight over the condition, their focus lies in preserving their health, maintaining function, and retaining control for as long as possible. Importantly, whilst other researchers might debate the use of military metaphors in chronic illness descriptions [50], we advocate for using the language employed by PwP. For instance, one attendee said that by joining the class they [group members] were:

Not letting it take over our lives … Fighting back … we are not passive … not letting it take over our lives … we are fighting against it. [Class attendee #5]

Even though not all of the attendees were habitual exercisers prior to their engagement with this CEBP, participants echoed the idea that fighting back is a proactive mindset and that exercise is a means of resistance to the progressive nature of the condition.

Moreover, participants stated that engaging with the current exercise programme encouraged them to take part in other group activities (e.g., boxing, choir singing, comedy). Thus, PD-specific CBEPs have great potential to encourage PwP to join other community groups. Nonetheless, research shows that continual support is needed, particularly the care and management of disease-related apathy or the reduced initiative to engage with exercise [51].

***Acceptance and adjustment.*** Attendees spoke about how living with Parkinson’s required adjustment and acceptance that their condition will worsen over time and the need to remain positive. Some found it hard to accept their diagnosis and its implications (also discussed under ‘Family encouragement and support’). One partner described her husband’s initially low motivation to exercise, attributing it to his negative outlook upon diagnosis:

“Because he was quite negative when he first knew, which I suppose is quite understandable … it’s a bit of … especially when he knew is going to get worse as time went on. . so he was very negative, it was 24/7 almost sitting on the sofa sleeping … and of course, it’s not very good for us either.” [Partner #2]

Attendees voiced mixed feelings about living with Parkinson’s. For example, one spoke about how it was important to maintain a positive outlook in order to cope with the condition:

I’m not progressing … at least I’m not … probably getting any better, but I’m not … I’m certainly not getting any worse … It’s [exercise] maintaining your health. And I think that’s an important thing. [Class attendee #2]

In contrast to this, another attendee, appeared to harbour some resentment at getting Parkinson’s when they had been a regular exerciser, although this does not seem to have discouraged them from continuing to exercise (or joining the class in the first place):

I have always been a member [of the gym] … I used to do karate, play rugby … Kept myself reasonably fit by going to the gym, but that didn’t stop me from getting Parkinson’s, though. [Class attendee #7]

***Family encouragement and support.*** It became clear that, for some attendees in the group, their partners played a key supporting role, and without their encouragement they perhaps would not have joined the class nor attended as often.

Despite the widely acknowledged benefits of exercise as medicine for managing health, it is not for everyone—nor are its benefits for individual health accepted by all [52]. In some instances, realising or accepting these benefits may take time. It is understandable that some attendees might have had reservations about joining the group (refer to the quote by Class attendee #7 under “Acceptance and Adjustment” ). For instance, one partner commented that, initially, her husband was reluctant to attend the class as he did not want to see others who were at a later stage of Parkinson’s (which has also been illustrated in a recently published qualitative study [17]. She described how she encouraged her husband to participate in the exercise class by suggesting that his involvement [in the project with the university] would help others:

I’ll be honest, [he said] he didn’t really want to come here … But I said: “You will not only be helping yourself, but you will also be helping others.” … And with that, he said: “Alright, I’ll go!” [Partner #2]

Partners’ encouragement and support were seen as important factors contributing to attendees’ motivation and prolonged involvement in the weekly exercise class. Motivation to attend the exercise class often varied, but participants described how structured supervised group exercise encouraged engagement, despite fluctuating motivation and mood. One attendee commented how his partner provided encouragement when he did not feel like going to the class:

That’s interesting you say that because my wife always asks me about 5 o’clock on a Tuesday … says, “are you going to Parkinson’s class tonight?” And if I say, I don’t know, it’s a bit cold and a bit miserable, I get a kick up the backside… [she says] “get yourself out there, you miserable being!” [Class attendee #1]

The lack of motivation to exercise outside of the class can be frustrating for those partners who also want to be physically active, and it would appear that they sometimes forgo their own physical activity goals to support their partners:

That’s something I can’t get mine [husband] to do … I would like … because I need to lose weight and I would like to go and do on walks, but … he cannot just see the point of just going for a walk, and I’ve … so, I’ve given up! [Partner #3]

Similar findings have been published previously calling for a more nuanced discussion about whether exercise truly is medicine for all [52, 53]. These views were echoed by other partners in the group who commented that not everyone with Parkinson’s in the class shares the same amount or type of motivation and/or wants to exercise—or takes part in the activities on offer to the local Parkinson’s network support group.

Some of these … some of the people with Parkinson’s are very self-motivated—very into beating Parkinson’s, finding the cure and, and what have you. My husband is one of the least motivated in that respect; he’s just doing what he’s got to do. [Partner #4]

***Helping others helps oneself.*** Partners employed various motivational strategies to encourage their loved ones’ participation in the class (see quote by Partner #2 under “Family Encouragement and Support”). Additionally, some attendees found their involvement in the research to be a compelling reason to participate. All class attendees actively participated in the periodic evaluations (described elsewhere) although their level of interest in the results varied. One attendee expressed, "It’s nice to be able to help other people as well, because from these studies, the feedback will be used to help other people, which is … you know … partly … the main of the exercise isn’t it, really?” [Class attendee #6].

Recent research underscores altruistic motivation in participating in community-based research for others’ benefit [54]. In this study, both attendees and partners discussed motivation to help researchers and other PwP. However, discerning whether participants are primarily motivated to selflessly help others, or whether their motivation is self-interested (or a combination of both) is challenging. As one attendee admitted, they were keen to try anything that is free, commenting that financial issues may influence the type of treatment sought to self-manage PD. This highlights that cost is a potential barrier to participation for certain demographics, such as older individuals and people with disabilities and/or long-term conditions [26]. Another class attendee (#5) mentioned that some classes “… cost a fortune … and [this one] costs half the price." Along these lines, one partner [Partner #4] admitted that "It [exercise] was just suggested that it might do some good, and you’ll get to a stage where you’ll give anything a go, really.”

### Theme 2: exercise is medicine for the mind and body

Class attendees and partners consistently emphasised that exercise provides a variety of benefits, particularly when conducted in a group setting with supportive instructors and student volunteers. While attendees and partners acknowledged the existence of potential side effects, they believed that the positive outcomes outweighed any negative responses. Some expressed a desire for more exercise but considering the reported side effects by others (such as fatigue, disturbed sleep patterns, muscle cramping, or feelings of exhaustion), an additional session in the week may not be suitable or provide additive benefit.

***Exercise as symptom management.*** Attendees emphasised the value of exercise for maintaining overall health and managing their Parkinson’s symptoms. They believed that exercise played a crucial role in managing both motor and non-motor symptoms, enabling PwP to take control over their condition [30].

If you don’t take this drive to do this exercise class you find the other non-motor symptoms of Parkinson’s … anxiety, all those sorts of horrible things … apathy … all creeping in on you, especially coming back to the dark nights and wintertime. If you make that effort to come along to the group exercise, I believe, I do believe in my heart, it’s much better for you and you will get a better quality of life for it. [Class attendee #3]

… this class is just as good as the medication, if not better. I said it’s part of my whole medication regime. [Class attendee #5]

***Challenging the discourse around exercise for PwP***. Class attendees and partners discussed the varying attitudes and recommendations that they received from healthcare professionals (HCPs) regarding exercise. While HCPs have a key role in supporting and facilitating the self-management of PwP [55], some class attendees were initially discouraged from engaging with high-intensity MM exercise and advised to stick to seated exercise:

There were very strong claims that … really strong exercise can actually put you back in your disease, but I … I don’t believe that … what I do believe is that you can hold it … [Class attendee #3]

While one attendee emphasised the importance of exercise for everyone saying that “We should be doing exercise whether we’ve got Parkinson’s or not!” [Class attendee #6], another attendee [#3] mentioned that they “… needed a focus for it” suggesting that exercise motives among the group differed. There were further discussions around experiences with healthcare professionals and stories focused on the lack of support for exercising with Parkinson’s.

I was told by a senior physiotherapist [within hospital] that we … we won’t ever run a Parkinson’s exercise class because it wouldn’t be supported. [Class attendee #3]

Similarly, another attendee recalled how a healthcare professional was sceptical about exercise for PwP.

“Well, you know, I [HCP] don’t think you want to go there … well because you don’t want to see what’s coming, do you?” When they say that, the first thing you want to know is: “What’s coming!” [Class attendee #2]

It is possible that such views discouraged more people from joining the project. Ellis et al. (2013) reported that low outcome expectations are an important perceived barrier to engaging in exercise for PwP [56]. However, despite the mixed messages and advice from HCPs regarding exercise as therapy, regular participation in the class appears to have positively influenced opinions and perceptions of exercise among some of the attendees:

You see how successful this has been, but erm … erm, the official … NHS class is just seat-based, and they walk around … [Class attendee #5]

Well, apart from Parkinson’s, surely the human body wasn’t designed to sit still all day, was it? If you do, everything will just seize up—surely you’ve got to keep moving! [Class attendee #2]

The class attendees appeared to be strong advocates of exercise (even though not everyone was positive about its protective benefits), there was acknowledgement that the attitudes of some HCPs are also beginning to change.

I was being told by professionals … being put off exercising. . not to exercise “oh, don’t do that you’ll hurt yourself! Don’t cycle, you’ll do your back in!” This is [healthcare professional] talk. And she’s totally different now … she’s accepted that exercise can offer people so much, and so much hope. [Class attendee #3]

Partners also recognised the benefits of exercise for PwP and expressed that more could be done to raise awareness and encourage self-referral to exercise programmes:

I think it’s definitely a good thing … it definitely does them good … perhaps the people need to hear about it more because if I hadn’t read the advert for this class in one of those little booklets that come through the letterbox, I wouldn’t have known about it. [Partner #1]

Participation in such opportunities tends to be initiated through the local Parkinson’s support network and, in consequence, active members appear to benefit from the relations. Communication appears to be a barrier to increased uptake among the local Parkinson’s community and as one partner commented, doctors are often the first point of contact in the diagnosis process and so could do more to promote adjunctive treatments:

Why don’t they put them [the adverts] in Doctors’ surgeries? You see everything else on the walls—what you can do or join! [Partner #2]

The Doctors purely act as a prescribing mechanism. They don’t really get involved … [Partner #4]

However, not all partners encountered resistance or poor communication from HCPs. As one partner explained, her husband was encouraged to participate in the exercise class following a referral from the local Parkinson’s nurse:

… she came to the house, and she told me about, you know, what I could [for us] get as a carer, and she told me all the things that I, we could join, you know, like this [class]. [Partner #2]

***It lifts the spirits.*** Class attendees highlighted that, as older adults, social isolation and loneliness are a concern, which are known risk factors for poor psychological health (e.g., depression and anxiety) and well-being [57]. Community-based groups provide intergenerational opportunities for social interaction, a need emphasised by both attendees and partners in this study, suggesting they could meet before and after the class. Attending regular exercise classes is perceived to provide purpose (i.e., something to look forward to in the week) for these attendees and an opportunity to make new friends, potentially alleviating the psychological effects of social isolation. Attendees also discussed how regular exercise has impacted their mood as well as their quality of life:

I just love to come here … I feel tired and worn out when I go back, but it’s a happy feeling. I’m so pleased with what I’m doing. From my wife’s point of view, there’s been a change … I was very down … not depressed … but er … very quiet … and I suppose everything was going around … what was going to happen to me, but no … since I’ve met everybody else here and watched everybody and just the interaction, I think, between the group … apart from these … the activities, I think the interaction and meeting everybody every week … yeah, I think it’s a brilliant idea. [Class attendee #2]

… we’ve all got to do something … being given the dreaded disease… you’ve got to fill your time up with happiness … the exercise is one very good step to filling up your happiness cup. You feel excited and active, more so than when you went in! [Class attendee #3]

Similar views were echoed among the group, with other attendees also commenting how they often felt better for exercise, even if it is a struggle to find the motivation to turn up to the class on a Tuesday evening.

I drag myself there sometimes and I come away feeling high. [Class attendee #7]

I have come out of this sort of … not depression … but this quiet stage that was in … I had so much going through my mind [sic] … now, that’s taken most of that away … the fact that we are doing this [exercise] has taken that worry. [Class attendee #2]

***Side effects and impact on daily living.*** When discussing the short-term side effects of exercise (i.e., in the hours/days afterwards), attendees commented that fatigue and disturbed sleep were side effects that sometimes appeared after the exercise class and could compromise body functionality for daily living (e.g., getting dressed the following morning). Nonetheless, they were keen to emphasise the importance of exercise in combating PD and believed in its benefits for their overall well-being:

I’ve got Parkinson’s dystonia, which is muscle cramping and things, and I can almost guarantee that either … I will have a muscle cramp in the legs or the arms after the exercise class. But I still come and do it because I’m still fighting back Parkinson’s … [pause] and I really believe that. [Class attendee #5]

… I’m not sure, but I know one of the effects, certainly one of the effects that I have, and I certainly know [Class attendee #5] has, because I’ve got emails from him at 3 o’clock in the morning, is disturbed sleep pattern. And the one thing I do not get when I leave here is … I do not get a good night’s sleep … and I don’t normally but I thought sometimes I’ve left here, and I’ve thought like I’m going to sleep well tonight, and I might do till about 3 o’clock, then I’m up and about downstairs making a cup of chocolate. [Class attendee #2]

Partners also highlighted the intensity and duration of fatigue, describing how it impacted their relationships and daily activities. As one partner described, her husband took some time to adapt to exercise, whilst another partner commented that her husband would do more in the session than at home, highlighting that the exercise class would leave him shattered and exhausted.

When he first came, he was absolutely shattered. You know, he could hardly walk to the car. But he seems … to be coping with it better now. I don’t have to push him so much now, I just have to say, “it’s Tuesday now,” whereas before I had to coax him along, but he seems to be more accepting of it now. [Partner 1#]

Well, it takes my husband between two and three hours to get out of bed in the morning… sometimes he adds that in as an excuse, “I can’t move, exercise yesterday.” But it doesn’t really impact him to that degree at all… I mean my husband will insist in the mornings that he can’t stand up without using his arms, but I’ve just watched him in there! [Partner #4]

Fatigue and impaired sleep are non-motor manifestations commonly observed in individuals with PD and have been found to be associated with each other [58]. Some partners expressed concerns about the timing of the exercise class (starts at 18:00 h), suggesting that it might contribute to the appearance of these side effects. However, it was acknowledged that the class was originally designed for the working-age group (i.e., after working hours) and has now included retired individuals:

There have also been concerns raised in the past about the timing of this class because a lot of people with Parkinson’s are too tired at this time of the day, or their medication wouldn’t last sufficiently for them to be able to attend this class. [Partner #4]

Despite acknowledging the side effects, attendees were keen to stress that exercise was an essential part of their medication regime and form of self-care. Furthermore, giving the attendees personalised feedback on their data from the routine physical function assessments appears to be received positively:

I had my assessment a few weeks ago … […] And I made significant improvements. I think if I hadn’t done these classes, I would have drifted away … head down, miserable, depressed … [Class attendee #6]

One attendee perceived exercise had had a profound impact on their life: “I’m healthier now than when I started” [Class attendee #3]. The impact on daily living was echoed by another attendee who said that thanks to the exercise “I can move my arm now and I couldn’t before” [Class attendee #4]. Altogether, attendees agreed that exercise had benefited either their physical, emotional, and/or their social well-being. However, it is important to recognise that alongside these positive impacts, there may be potential side effects or unintended consequences that need consideration and tailoring to ensure that the benefits outweigh any potential risks or challenges [59, 60].

### Theme 3: A community working together to promote exercise for Parkinson’s

Attendees and partners emphasised the group was more than people coming together—there was a sense of shared endeavour among class attendees, partners who supported the class, the instructors [and researchers], and student volunteers [61]. This project was perceived to be more than a partnership or transactional relationship whereby the attendees were doing something for research and the students and researchers were doing something for the local community—it was about everyone working together toward the same goal on a consistent basis, bound by interdependencies.

***We need each other.*** Class attendees, and non-exercising partners, frequently highlighted how group activities such as the exercise class provide an opportunity to interact with others and avoid being lonely (also noted in qualitative studies involving PwP [17, 62].

… once you retire you all sort of drift away, once you pack up work you haven’t got many friends, and this … a group … for exercise reasons, it’s camaraderie as well. [Class attendee #3]

It’s not just about the exercise, it’s about meeting as a group, again it’s a very important part of the situation we have found ourselves in … to become part of a group. [Class attendee #3]

Participants were not only able to share their individual experiences with other group members, but they were also able to learn about the disease through observation in the exercise classes and engagement with staff from the university. For this group, there appears to be a strong sense of affinity as attendees and partners are united by a shared experience/shared condition (also highlighted by Claesson and colleagues [17] and other groups [63, 64]).

It’s nice to meet other people … fellow sufferers in other words … you know, different levels … some people are quite badly affected, some people are less badly affected. But it’s nice to mix amongst them. Ask each other questions and get feedback from them. It’s quite helpful, I think… Gives you a chance to chat to people, and pick up their experiences, which is … and the empathy. . quite powerful, that. [Class attendee #6]

I’d miss all this … it’s quite a strong bond [we have] actually… [Class attendee #7]

Scheduled group exercise can positively influence motivation and adherence over time, as well as exercisers’ affective experiences [65], by bringing people together to achieve shared goals:

That’s the trouble … you don’t see me going to [the gym] … [I] don’t go anymore … [I get] bored. It’s me on my own. You know, for me, I need a group … a team, a team. [Class attendee #3]

The various physiotherapists have given [...] exercises over the year and the pieces of paper are probably gathering dust in the magazine rack somewhere, but he will come and do this! [Partner #4]

***A class for everybody***. In a group environment, PwP can face social challenges, including anxiety about identifying with others at more advanced stages of PD [66]. Social comparisons can be overwhelming and have a negative impact on dropout rates, although in some cases they may actually increase confidence and motivation if participants perceive they are performing relative to others with more severe symptoms [67]. Partners believed the group format provided an accessible and inclusive exercise environment without fear of judgement:

… I suppose he doesn’t like to stand out … in front of, you know, like … because everybody else is doing an exercise he’s happy with it … if they all did it in turns, they all did it in turns or something, he probably wouldn’t want to stand out in the crowd, so to speak … [Partner #1]

As one partner commented, engagement with supervised group exercise may be due to the fact that it promotes greater autonomy support than prescriptive exercise interventions offered by HCPs. Group exercise allows attendees with different motivation levels to support one another but also work independently.

Yes, it’s not me telling him … because if he comes home with a piece of paper [from the physiotherapist] the only person that’s going to make him do it is me because he’s not self-motivated. [Partner #4]

***Supportive instructors and student volunteers make a difference.*** Attendees were keen to highlight how the instructors and student volunteers support motivation to exercise by varying the exercise routine, which maintains interest and enjoyment, as well as challenging attendees to work vigorously.

Partners did not comment much on the class itself as they said they do not know what goes on, but they did discuss how their loved ones would comment on the interactions with others rather than the exercises, particularly focusing on the effect that positive reinforcement has on motivation.

The instructors have made him more positive … he comes to the class every week … it’s quite wonderful … they say well done … he loves to hear that. [Partner #2]

Verbal encouragement from the instructors and helpers have been shown to support motivation and confidence [68]. Ellis et al. (2013) suggested that cognitive-behavioural strategies such as goal setting and feedback could be important targets for facilitating behavioural change in PwP [56]. Although our findings do not indicate which strategies are most effective, they do suggest that motivational support and positive reinforcement from the instructors and student volunteers promotes a perceived supportive environment to exercise, a finding evident in Rossi et al. (2018).

The class attendees described how they enjoyed the social interaction with the students during the exercise class and one attendee said “I think the students really enjoy it as well, which helps” [Class attendee #7]. One of the partners also commented on the role of the students, “… the number of volunteers is incredible, and the one-to-one ratio, yes it’s very good, I think it’s probably three-to-one tonight!” [Partner #4].

However, a drawback is that during the summer months student support drops off. Some of the attendees had formed close social bonds with the students and were sad to see students leave:

I think the fact that the students are so good and friendly. It’s a shame that when it comes to the end of the year, and they disappear. Then you start with another group, and they are all just as good as the last lot. . and you work your way around. [Class attendee #4]

## Discussion

This qualitative study has captured attendees’ and partners’ views and experiences of a group exercise class for PwP supported by a community-university collaboration. Our findings reveal differences from previous research on community-based exercise groups [17, 34], particularly regarding reasons for joining the class (e.g., to benefit others) and mixed attitudes among professionals towards exercise as a therapeutic intervention (e.g., exercise can provide ancillary benefits beyond physical fitness versus PwP should not be exercising). The exercise class was perceived positively by attendees and partners, fostering a sense of community and fellowship as participants shared stories of collective coping and positive social interactions. Attending and supporting participation in community-university supported programmes for PwP appears to support self-management as well as foster an ethos of working together as a team. Even for those who are less motivated and empowered to “fight back”, exercise can provide a positive experience—providing it has a focus (exercising for the sake of it does not appear to be enough). When delivered in a group circuit-style format, supervised by instructors, it appears to offer additional benefits, including accessibility and inclusivity. Thus, the social support provided by the group meetings appears to be a key factor contributing to the class’s long-term success. Attendees and partners formed friendships and acknowledged the contributions of instructors and student volunteers, suggesting that the intergenerational aspect of the collaboration plays a crucial role.

It is easy to assume that individuals who volunteer for exercise interventions or join community-based initiatives are motivated to exercise. However, we found that motives for exercising differed. Some participants were already enthusiastic about exercise or were keen to interact with others, while others were initially reluctant to take part. Class attendees and partners highlighted how they or their partners were encouraged to join the class for the potential benefits to themselves and others and suggested that the information and advice they received from HCPs about exercise participation varied. Formal support for exercise is key to helping PwP start and adhere to suitable exercise programmes [69]. There was also agreement across both focus groups that more could be done to promote exercise for PwP and to explore a range of complementary non-pharmacological treatments, something that is being considered by various lines of research [70, 71]. Overall, class participants expressed a willingness to devote time and effort to health research activities but would like to see more evidence that the findings will be used to benefit others, including the local PD community and HCPs. Regular feedback and dissemination of research findings to the community is therefore key to sustaining interest in such activities.

Another important part of exercising in a community-based class is the beneficial effect of combining a philosophy of inclusiveness with a programme that seeks to enhance physical activity and social engagement. Our data reinforces previous work which suggests that, once involved with CBEPs, attendees perceive a sense of social belonging and feel less isolated and lonely [19]. This is particularly important for PwP, who might use exercise settings as social networks [20], something that is critical for healthy ageing (i.e., postponing cognitive decline and neurodegeneration) and reducing the risk of developing dementia [72]. Although the community setting in which the exercise class was delivered provides both class attendees and partners with space to interact, some participants expressed that they would be keen for more opportunities for interaction outside of the class hours and expressed interest in exploring ways to extend social interactions, through organised events or gatherings.

Despite the high attendance recorded in this study, it is important to reinforce a key finding from our data that motivation for exercise can be influenced by fluctuating moods and physical functionality [73]. To address these challenges, a multifaceted approach was employed, leveraging familial involvement, instructor and student volunteer support, and motivational encouragement. These approaches tapped into class attendees’ perceptions of belonging to a group (and working together), their meaningful contribution to the Parkinson’s disease community (benefitting research and future PwP), as well as the challenge posed by the exercises. In terms of the delivery and accessibility of the exercise class, factors associated with prolonged involvement hinged on providing a supportive environment that promotes inclusivity (making exercise accessible to PwP at all stages of disease severity), creating fun and challenging exercises to maintain interest and alleviate boredom, providing encouragement to build confidence, and nurturing friendships and relationships between class attendees, partners, student volunteers, and instructors. The high level of supervision (with a favourable 1:2 ratio of instructor and student volunteers to attendees) and involvement of the university were instrumental to the success of the project, but reliance on student volunteers outside term time presents challenges. While our model of service delivery has yielded promising outcomes, its potential replication in other community settings warrants further exploration.

Finally, based on the insights gained from this study, we offer several pragmatic suggestions to assist those involved in delivering CBEPs for individuals with Parkinson’s disease (PwP) and their partners (Table 2) to decide which tools or approaches might be more useful. These recommendations, developed as an outcome of the present qualitative research findings, take into consideration the multifaceted nature of participants’ experiences and motivations, as well as the benefits and challenges associated with such programmes.

**Table 2:** Exercise for PD: Recommendations for building and sustaining community-based interventions

Recommendations	Description
1. Tailored Engagement	Try to develop outreach strategies that consider the diverse motivations and concerns of potential participants, particularly the specific needs of individuals who are hesitant to engage due to factors such as social isolation or symptom exacerbation fears. For example, offering information sessions can help address concerns and highlight the benefits of exercise for both physical ability and emotional well-being.
2. Socialisation Support	Fostering a supportive environment that goes beyond the exercise component can help build relationships. Intergenerational programmes that bring together students and older adults provides positive opportunities for social interaction and community building. We encourage trying to develop meaningful social connections by incorporating structured opportunities for participants to interact before and after exercise sessions.
3. Motivational Strategies	Implementing cognitive-behavioural strategies such as goal setting, feedback, and positive reinforcement during exercise sessions motivates participants. Instructors and student volunteers play a crucial role in providing individualised encouragement to boost participants’ confidence and motivation.
4. Inclusive and Structured Programming	Try to create structured exercise programmes that accommodate participants at different stages of PD progression. Providing variations of and adaptations to exercises helps cater for varying levels of mobility and ability. Mixing up the routines helps to reduce boredom. Ensure that the exercise routines are challenging yet attainable for all participants by providing a range of exercise options, including high-intensity activities. Incorporate flexibility and progression into the exercise routines to accommodate participants’ changing needs over time.
5. Collaboration with Healthcare Professionals	Identify opportunities to collaborate with healthcare professionals (HCPs) to promote the benefits of community-based exercise programmes as an integral part of care and encourage HCPs to discuss exercise options with patients during diagnosis and follow-up appointments. Facilitate open communication between programme organisers and healthcare providers to encourage referrals and support (e.g., by providing HCPs with up-to-date information on the class outcomes for PwP).
6. Consistent Availability	Try to consider ways to address challenges related to programme availability/accessibility by exploring options for additional class sessions or alternative time slots to accommodate different schedules. Maintain a consistent level of support, including instructors and reliable student volunteers, throughout the year to ensure safety, continuity, and participant engagement.
7. Routine Monitoring and Evaluation	This involves both qualitative and quantitative longitudinal approaches for evaluation. For example, evaluating the impact of participation on physical and cognitive function, emotional well-being, quality of life, and service satisfaction over extended periods. These findings can optimise programme delivery based on participants’ evolving needs and strengthen participants’ engagement.

### Strengths, limitations, and future research directions

To enhance qualitative rigour, we considered several recommendations and strategies outlined by Smith and McGannon (2018) [74]. In particular, we engaged in continuous self-reflection, considering how individual roles and perspectives within the research team might influence the interpretation of the data (two of the authors had dual roles [as researchers and instructors] having also delivered the exercise sessions, and were involved in recruitment). Whilst it is important to consider how such embeddedness might influence the interpretation of the data, being close to the data offers several advantages such as being able to provide contextual knowledge and share experiences.

The limitations of this study are centred around the homogeneity and socio-demographic and clinical characteristics of attendees who took part in the exercise class. All PwP who participated in this focus group study were male, although this may not be all that surprising as men are reported to be at greater risk for Parkinson’s [75, 76]. Given the predominately male class membership (83%), the views may only represent the views of a subsample of attendees in the class, and future research should include the perspectives of women. Additionally, interviewing student volunteers, former class attendees, or those from the local Parkinson’s network support group who actively chose not to join the exercise class would provide valuable insights, although accessing them poses challenges.

It is important to note that the level of support within this exercise programme is unique. Such support is unlikely to be available elsewhere, which does pose a resource challenge to deliver a similar class experience. For example, class attendees commented that they would like a second class each week; however, challenges include finding instructors, student volunteer helpers to maintain a similar volunteer-to-attendee ratio, and the space and cost to run the class.

## Conclusions

This study explores class attendees and partners’ perceptions of a community-based group exercise class for Parkinson’s within the context of a community-university partnership model. Participating to help others is a potentially powerful motivational strategy to encourage more members of the community to participate in research-supported initiatives. However, more could be done to promote partnerships and share resources in the community, particularly between clinicians and researchers. The class provides participants with a sense of companionship, meaningful social connections, fellowship and a perceived positive affective experience—psychosocial benefits that also extend to partners who come along to support their loved ones, and that are important for exercise adherence and achieving positive health outcomes. These insights can assist relevant parties in delivering tailored and effective exercise interventions for PwP, addressing the multifaceted needs of this population.

## Data Availability

The transcripts of the focus group interviews used are available from the corresponding author on reasonable request.
